# Rapid multiplexed nanopore amplicon sequencing to distinguish *Plasmodium falciparum* recrudescence from new infection in antimalarial drug trials

**DOI:** 10.1038/s41598-025-20925-7

**Published:** 2025-10-22

**Authors:** Aurel Holzschuh, Anita Lerch, Christian Nsanzabana

**Affiliations:** 1 Swiss Tropical and Public Health Institute, Kreuzstrasse 2, 4123 Allschwil, Switzerland; 2 University of Basel, Basel, Switzerland; 3 Department of Biological Sciences, Eck Institute for Global Health, University of Notre Dame, Notre Dame, IN USA

**Keywords:** Malaria, *Plasmodium falciparum*, Molecular correction, Amplicon sequencing, Nanopore sequencing, Clinical trial, Therapeutic efficacy study, Genotyping, Genetic diversity, Drug resistance, Biological techniques, Biotechnology, Diseases, Microbiology

## Abstract

Evaluation of antimalarial drug efficacy against *Plasmodium falciparum* requires molecular correction to distinguish recrudescence from new infections by comparing parasite genotypes before treatment and in recurrent infections. We aimed to assess the utility of nanopore sequencing in providing rapid corrected drug efficacy estimates, particularly in patients from high-transmission settings where polyclonal infections are common. We optimized a multiplexed AmpSeq panel targeting six microhaplotypes to achieve high and uniform coverage. The assay’s sensitivity and specificity for detecting minority clones in polyclonal infections and its reproducibility were evaluated using a range of mixtures of four *P. falciparum* laboratory strains at defined ratios. Genetic diversity across the microhaplotype markers was assessed using 20 paired patient samples from a clinical trial. A custom bioinformatics workflow was used to infer haplotypes from polyclonal infections, including minority clones, applying rigorous cutoff criteria for accurate haplotype calling. The nanopore AmpSeq assay provided uniform and high read coverage across all six microhaplotype markers in both laboratory strain mixtures and patient samples. It demonstrated high sensitivity in detecting minority clones (as low as 1:100:100:100 in the 3D7:K1:HB3:FCB1 strain mixtures), high specificity (false-positive haplotypes < 0.01%), and robust reproducibility (intra-assay: 98%; inter-assay: 97%). The markers exhibited high genetic diversity (highest for *cpmp* with H_E_=0.99 and 28 unique haplotypes). Across the paired patient samples, the assay consistently distinguished recrudescence from new infections in 17/20 cases (85%) for all six markers. Our study demonstrates the feasibility and robustness of nanopore AmpSeq for distinguishing recrudescence from new infections in paired samples from clinical drug trials, offering a promising tool to obtain rapid corrected estimates of drug failure.

**Supplementary Information:**

The online version contains supplementary material available at 10.1038/s41598-025-20925-7.

## Introduction

The emergence of artemisinin-resistant *Plasmodium falciparum* parasites and subsequent treatment failure of artemisinin-based combination therapies (ACTs) are a significant public health concern threatening global efforts to control and eliminate malaria. Delayed clearance following treatment with artemisinin derivatives, also referred to as artemisinin partial resistance (ART-R), was first described in 2008^1^ and was initially confined to Southeast Asia. However, the recent independent emergence of ART-R in several countries from East and Horn of Africa^2–5^ is challenging ACT and malaria control in sub-Saharan Africa. Widespread ART-R combined with resistance to partner drugs (i.e., ACT failure) in sub-Saharan Africa would have a catastrophic public health impact, as the region bears the heaviest malaria burden^6^. Regular reporting of drug efficacy across sites in Africa is essential, particularly in areas where *P. falciparum kelch13* mutations have been reported, the key mediator of ART-R^7^.

Therapeutic efficacy studies (TES), conducted every 2–3 years in malaria-endemic regions, are considered the gold standard to assess antimalarial drug efficacy. In these trials, patients with microscopy-confirmed *P. falciparum* infection are treated with antimalarials and followed up at regular intervals to evaluate their clinical and parasitological response. During follow-up, some patients may experience recurrent parasitemia, which can arise from recrudescence - the original infection not being fully cleared (true treatment failure)—or from a new infection (new mosquito inoculation), or a mixture of both. To distinguish between these, molecular correction, also referred to as PCR-correction, is performed^8,9^. This involves genotyping parasites from paired blood samples collected on day zero (before treatment) and at the day of treatment failure, to distinguish recrudescence from new infection. The therapeutic efficacy (genotype-corrected efficacy) estimate, the primary outcome of TES, reflects the ability of the treatment to fully clear initial infections and potentially provide prophylactic effects by preventing new infections^8^. Various genotyping methods and molecular markers are in use, along with differing algorithms for results interpretation^9,10^, making it challenging to directly compare drug efficacy estimates across studies and regions. The World Health Organization (WHO) currently recommends genotyping three size polymorphic markers - *P. falciparum* merozoite surface protein 1 and 2 (*msp1* and *msp2*) and microsatellites—using capillary electrophoresis^8^.

Recent advances in next-generation sequencing (NGS) techniques have facilitated the development of targeted amplicon sequencing (AmpSeq) protocols of highly polymorphic markers for genotyping malaria parasites in clinical trials of antimalarial drugs^11–13^. By including short, highly diverse loci with abundant single nucleotide polymorphisms (SNPs), such high heterozygosity microhaplotypes can greatly improve discrimination between different parasite strains^14–18^. Detection of minority clones is possible at frequencies as low as 0.1% in polyclonal infections^11,12,18,19^, and data can be analyzed using inferential methods considering allele sharing probabilities based on population frequencies^20,21^.

Nanopore sequencing is increasingly used to generate genetic data on malaria parasites, including in endemic regions, for applications such as detecting antimalarial drug resistance markers, identifying variations in vaccine targets, characterizing *hrp2/3* gene deletions, and studying parasite population dynamics^22–28^. Oxford Nanopore Technologies (ONT) offers a low-cost, portable, and scalable alternative to traditional sequencing methods, potentially addressing the need to expand and decentralize sequencing capacity in resource-limited settings^29,30^. Workflows with ONT are straightforward, offer fast turnaround times, and are more easily deployed in endemic settings than other platforms^23,24,26,29^. Thus, nanopore AmpSeq could provide rapid corrected estimates of drug efficacy, for example if a TES is needed in response to rapid emergence of potentially drug-resistant parasites. While nanopore sequencing used in previous malaria studies primarily focused on genomic surveillance^23,24,26^, few studies have used nanopore AmpSeq to explore within-host diversity and detection of minority clones in polyclonal infections^26^.

Here, we evaluated the feasibility of a nanopore AmpSeq assay for genotyping *P. falciparum* to distinguish recrudescence from new infection in clinical trials by assessing sensitivity, specificity, robustness and genetic marker diversity using the current latest ONT chemistry (kit 14/R10.4.1 flow cells) and comparing genotyping results to data generated with Illumina AmpSeq^13^. We used a multiplex AmpSeq panel targeting short, highly informative microhaplotype loci that are amenable to multiplexing, exhibit high amplification efficiency, and are compatible with streamlined, field-adaptable workflows.

## Methods

### Laboratory strain mixtures

Laboratory *P. falciparum* strain mixtures were prepared as previously described^13^. Briefly, parasitemia of synchronized ring-stage parasite strains 3D7, K1, HB3, and FCB1 was quantified by qPCR. The four strains were then combined in mixtures ratios ranging from 1:1:1:1 to 1:100:100:100 and diluted in human DNA from malaria-negative donors to mimic clinical samples. The concentration of the minority clone was always 10 parasites/µL. Twenty-four different ratios were used for this study, and each mixture was tested in triplicate in each of the two sequencing runs. Parasitemia and ratios of all strain mixtures is available in Supplementary Table 1.

### Patient samples

Twenty well-characterized paired frozen whole blood samples (*n* = 40) from patients with recurrent infections were used from a previously conducted randomized, phase 2 clinical trial evaluating the efficacy and safety of cipargamin in adults with uncomplicated *P. falciparum* malaria in Mali, Gabon, Ghana, Uganda, and Rwanda. The protocol and all amendments were reviewed by the Independent Ethics Committee or Institutional Review Board for each center. All approvals were obtained in 2017 and 2018. A list of the institutions is provided in the Supplementary Table 2. The trial was conducted according to International Conference on Harmonization (ICH) E6 Guidelines for Good Clinical Practice. Informed consent was obtained from all subjects and/or their legal guardian(s). The trial is registered with ClinicalTrials.gov (NCT03334747). The trial was conducted between November 16, 2017, and November 23, 2019. Details of the study protocol and patient recruitment have been published elsewhere^31–33^. Parasite counts (Giemsa-stained thick and thin films) were made per 200 white blood cells (or if the count was < 100 parasites, counting was continued for up to 500 white blood cells). Parasitemia of the twenty paired samples ranged from 31 to 33,930 parasites/µL (Supplementary Table 3). The samples had previously been analyzed as part of a comparative evaluation of genotyping methods, including AmpSeq using the Illumina platform^13^.

### Multiplex PCR

Six polymorphic microhaplotype loci (*ama1*, *celtos*, *cpmp*, *cpp*, *csp*, and *surfin1.1*; Supplementary Table 4) were selected based on previously demonstrated high genetic diversity and discriminatory power^14,15,17,26^. Additionally, the genetic diversity of the corresponding genes has been extensively characterized across multiple studies^11,12,14,18,34–36^. We leveraged an existing 6-plex PCR panel targeting these loci^26^ and optimized it to ensure uniform amplification across all targets. All six amplicons use previously published primer sequences^14,15,26^. Details on primer pool concentrations and reaction conditions are provided in Supplementary Tables 5–7. An overview of A + T content and homopolymer regions (≥ 3 bases) within each amplicon is shown in Supplementary Fig. 1.

### Library Preparation and sequencing

Nanopore sequencing was performed using the Native Barcoding Kit 96 V14 (SQK-NBD114.96) on the MinION Mk1C platform with MinKNOW software (v24.06.15) and R10.4.1 flow cells according to the manufacturer’s instructions with some modifications (Supplementary Text). Each run included three negative controls, consisting of nuclease-free water, which underwent the entire workflow, including PCR and library preparation. A positive control (strain FCB1) was included alongside the paired patient samples for quality control. For each sequencing run, the target sequencing depth was approximately 25,000 reads per marker per sample, or 150,000 reads total, to compensate for downstream filtering of low-quality reads. Sequencing was stopped once the desired number of reads was reached. An overview of the complete workflow is shown in Supplementary Fig. 2 and a laboratory protocol, including materials and primer sequences, is available online at protocols.io (https://www.protocols.io/) as “Rapid multiplexed nanopore amplicon sequencing of Plasmodium falciparum microhaplotype loci”^37^.

### Bioinformatics pipeline

Raw nanopore data was simplex basecalled and double-ended demultiplexed with dorado (v0.8.2) using the super-accurate (sup) model with the minimum q-score for passing reads set to 20 (accuracy of ≥ 99%) to minimize erroneous reads, as previously described (Supplementary Text)^26^. Haplotypes were inferred using R packages HaplotypR (v0.5)^11^ and DADA2 (v1.26.0)^38^ as previously described with minor modifications (https://github.com/lerch-a/HaplotypR/releases/tag/v0.5; Supplementary Text)^26^. To remove false-positive haplotypes and artefacts, a number of cutoffs were implemented: (1) each marker required a minimum of 1000 total reads per sample, (2) haplotype calls required a minimum coverage of ≥ 20 reads per haplotype, and (3) a within-sample allele frequency (WSAF) of ≥ 0.1%. Additionally, reads with ambiguous base calls (e.g., N) and incorrect sequence length (e.g., to remove amplification and/or sequencing errors caused by homopolymer-rich regions) were removed. An overview of the bioinformatics workflow is shown in Supplementary Fig. 3.

### Data analysis

The limit of detection (LOD) of minority clones was defined as the lowest ratio of laboratory strains having at least two out of three replicates positive. To assess the intra-assay variability, we calculated the percentage of samples that returned the three technical replicates with the same result, and the inter-assay variability as the percentage of pairs of technical replicates giving the same LOD between two runs. Two algorithms were used to classify genotyping results for the six microhaplotype markers. The WHO algorithm defines sample pairs as recrudescent when all markers used show recrudescence^8^, while the two out of three algorithm classifies sample pairs as recrudescent when the majority of markers used (i.e., a minimum of 4/6) show recrudescence^39^. Allelic frequency was assessed by calculating the number of times, each haplotype occurred in all pre-treatment samples divided by the number of times all haplotypes occurred in all pre-treatment samples. Heterozygosity (H_E_) for each microhaplotype marker, the probability that two alleles will be different, in the twenty pre-treatment patient samples was determined using the equation $\:{\text{H}}_{\text{E}}=\:\frac{n}{n-1}[1-\:\sum\:{p}_{i}^{2}]$, where *n* = the number of samples analyzed, *p*_*i*_ = the allele frequency of the *i*^th^ allele in the population. The combined probability of discerning two clones using any of the microhaplotypes was estimated using $\:{P}_{diff}=1-\:\prod\:_{i=1}^{n}(1-{H}_{E,\:i}$), where *H*_*E, i*_ = expected heterozygosity for microhaplotype *i*. Multiplicity of infection (MOI) was estimated in two ways: (i) naively, where MOI was determined by the locus with the greatest number of detected alleles, and (ii) using SNP data from the microhaplotypes to estimate MOI using THE REAL McCOIL^40^, run in categorical method. Pairwise genetic relatedness based on identity-by-descent (IBD) in the paired patient samples was estimated using Dcifer (v1.2.1) with default settings, which also provides 95% confidence intervals (95%CI) via likelihood ratios^21^.

## Results

### Sequencing quality and coverage

**Fig. 1:**
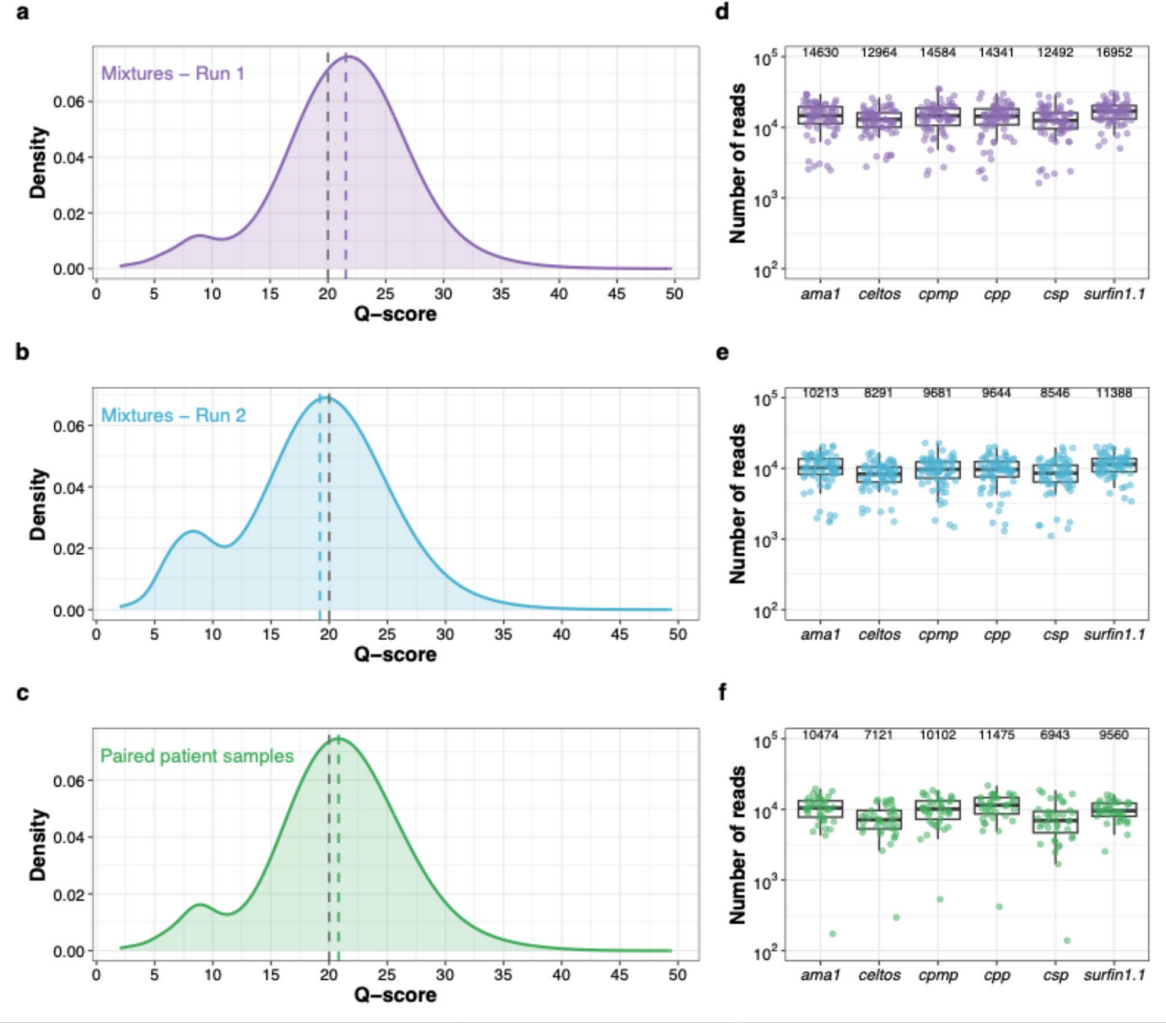
Quality metrics and read coverage profiles of three MinION sequencing runs. Distribution of q-scores for the control mixtures (**a**) run 1 (*n* = 72, median q-score 21.6)) and (**b**) run 2 (*n* = 72, median q-score 19.2), and (**c**) paired patient samples (*n* = 40, median q-score 20.8). The dotted grey line indicates the minimum q-score of Q20 for passing reads and the dotted colored lines indicate the median q-scores of each sequencing run. Coverage profiles of amplicon targets of run 1 (**d**) and run 2 (**e**) for the control mixtures and paired patient samples (**f**). Each dot represents a sample. The y-axis shows number of reads (log10) covering each amplicon target per sample for each of the three MinION runs. In all three figures, positive and negative controls were excluded. Median coverage for all amplicons is indicated on top of the graph. The box bounds the IQR divided by the median, and Tukey-style whiskers extend to a maximum of 1.5 × IQR beyond the box.

We sequenced triplicates of 24 control mixtures across two independent MinION runs (*n* = 72 in each run), and 20 paired patient samples (*n* = 40) in a third run. All samples were successfully amplified and sequenced. The q-scores of reads from both control mixtures and patient samples were comparable across all three sequencing runs (Fig. 1a-c, Supplementary Table 8). PCR optimization (Supplementary Fig. 4, Supplementary Table 9) improved coverage uniformity across all amplicons in both control mixtures (Fig. 1d-e) and patient samples (Fig. 1f). After filtering out reads with q-scores ≤ Q20, the median coverage across control mixtures was 85,789× per sample (interquartile range (IQR): 68,172×–107,275×) in run 1 and 58,591× (IQR: 45,819×–73,139×) in run 2. The median coverage for the 20 paired patient samples was 55,105× (IQR: 40,435×–72,904×). The median fold-difference in coverage among amplicons was 1.4 (IQR: 1.3–1.7) in run 1 and 1.5 (IQR: 1.3–1.7) in run 2 of control mixtures, and 1.7 (IQR: 1.5–2.2) in patient samples.

### Specificity, limit of detection, and reproducibility using control mixtures

Haplotype calling showed high specificity. Only one false-positive haplotype passed the cutoff criteria (≥ 0.1% WSAF and ≥ 20 reads) in a single replicate (sample S11) for marker *surfin1.1* at 1.02% WSAF (Supplementary Fig. 5). In control mixtures containing only one of the four *P. falciparum* strains (S01–S04), trace contaminant haplotypes from other samples were observed in 19/92 instances in run 1 (median reads: 2, range: 2–5) and 15/88 in run 2 (median reads: 2, range: 2–4), all below the defined cutoff. Additional false-positive haplotypes, likely due to amplification or sequencing error, occurred in 0.28% of amplicon–sample instances in run 1 (4/1,415; median 9 reads, range 7–229) and 0.07% in run 2 (1/1,408; 12 reads). Overall, contaminant or false-positive haplotypes represented < 0.01% of total reads (302/6,133,887 in run 1; 50/4,160,477 in run 2), with all but one occurring below 0.1% WSAF. Based on these results, a threshold of ≥ 20 reads and WSAF ≥ 0.1% was selected to minimize false positives while maintaining sensitivity. No marker in any negative control (*n* = 9 across three runs) exceeded 12 reads, precluding haplotype calling.

**Fig. 2:**
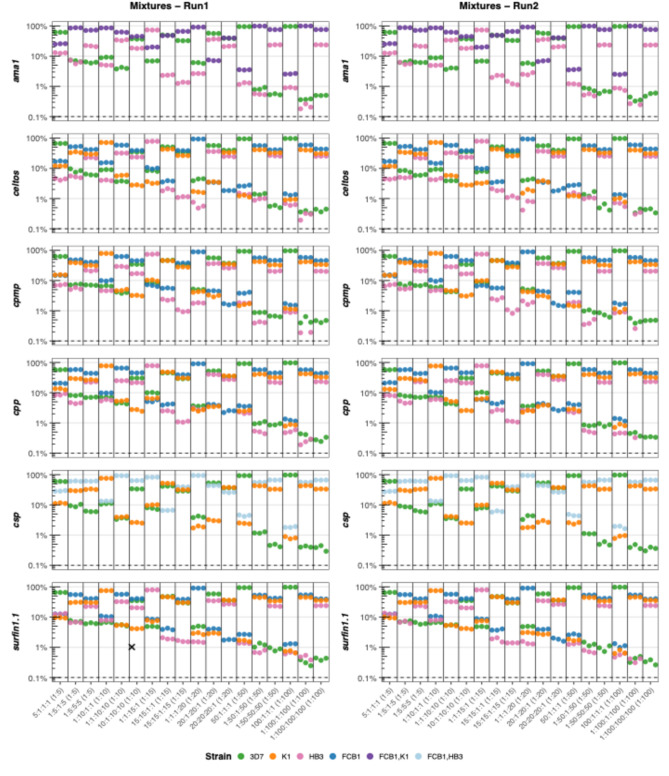
Limit of detection (LOD) for minority clones in control mixtures. Haplotypes detected in the control mixtures of four *P. falciparum* strains across the six microhaplotypes in Run1 (left panel) and Run2 (right panel). Every control mixture was sequenced in triplicate in both runs. The ratios represent the ratio of the four *P. falciparum* laboratory strains assessed in this study: 3D7:K1:HB3:FCB1, with the concentration of the minority clone always being 10 parasites/µL. The ratios in parentheses are for markers *ama1* and *csp*, as two strains had identical alleles (FCB1 and K1 for *ama1*; FCB1 and HB3 for *csp*) and LOD could only be determined for two strains instead of four. Y-axis: proportion of reads (log10). Dashed horizontal lines: WSAF cut-off for minority clones set at 0.1% of total reads. The black cross indicates a false-positive haplotype.

The LOD for minority clones was consistent across markers (Fig. 2, Supplementary Table 10). For two microhaplotypes, the LOD could not be determined for all four control mixtures due to identical alleles in *ama1* (FCB1 and K1) and *csp* (FCB1 and HB3). Detection of minority clones for these markers was assessed using only the strains with distinguishable alleles (3D7 and HB3 for *ama1*; 3D7 and K1 for *csp*), when one strain was in majority and the other in minority. At a WSAF of ≥ 0.1% in both runs, all six markers reliably detected minority clones even when majority clones were present at a 100-fold higher concentration (1:100:100:100), corresponding to a minority clone WSAF of 0.3% (Fig. 2, Supplementary Table 10). The assay also demonstrated robust performance and high sensitivity at low parasite density; in control mixture S05, containing all four strains at 10 parasites/µL each (40 parasites/µL total), all strains were consistently amplified and detected across all replicates in both sequencing runs. All markers exhibited high intra-assay and inter-assay reproducibility, ranging from 94.7% to 100% (Supplementary Fig. 6a). A strong correlation between expected and observed WSAF was observed in the control mixtures in both sequencing runs (R^2^ = 0.97, Supplementary Fig. 6b).

### Haplotype diversity and allelic frequency in paired patient samples

**Fig. 3:**
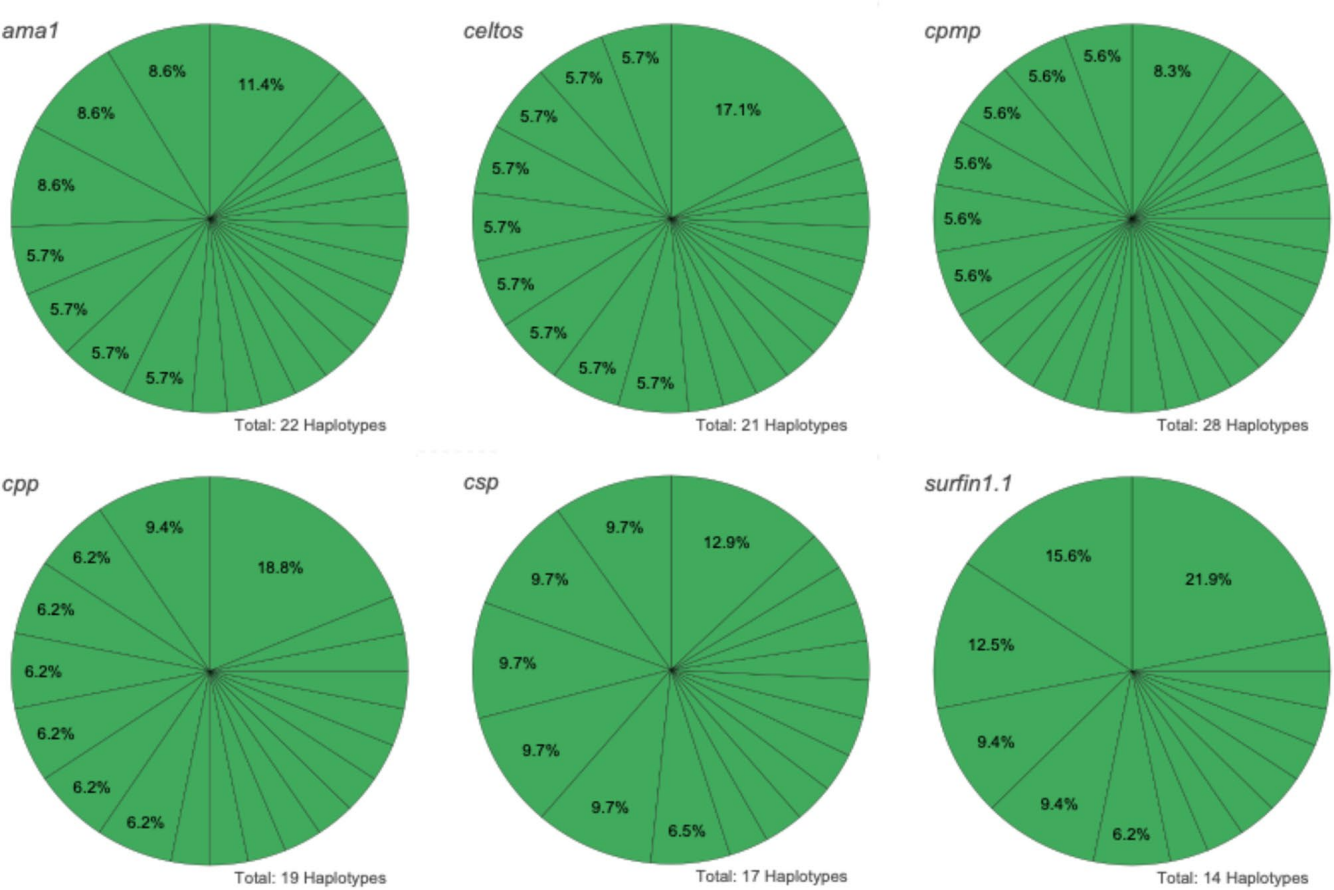
Diversity of the six microhaplotypes in the 20 pre-treatment patient samples. Each pie chart shows the distribution of haplotypes for each marker across the pre-treatment patient sample pool (*n* = 20). The total number of haplotypes found is shown at the bottom right of each microhaplotype pie chart. Haplotype frequencies greater than 5% are indicated.

The genetic diversity of the 6 microhaplotype markers was assessed in the 20 pre-treatment samples. Haplotype diversity, defined as the number of distinct haplotypes, was high but varied across the six microhaplotypes (median: 20, range: 14–28; Fig. 3; Table 1). Marker *cpmp* exhibited the highest diversity, while *surfin1.1* showed the lowest number of distinct haplotypes. Allelic frequencies also varied by marker (Fig. 3). For *cpmp*, no haplotype had an allelic frequency > 10%. Markers *ama1*, *cpp* and *csp* each had one haplotype with allelic frequencies > 10%, with no clearly dominant haplotype. In contrast, *celtos* had one haplotype at a frequency of > 15% and *surfin1.1* had three dominant haplotypes, collectively accounting for over 50% of all reads (Fig. 3). The number of SNPs ranged from 13 for *celtos* and *csp* to 39 for *cpmp* (Table 1). SNP frequencies and positions are shown in Supplementary Fig. 7 and Supplementary Table 11. The discriminatory power (i.e., the probability that two alleles will be different; H_E_) was > 0.9 for all markers (Table 1). The combined probability of discerning two clones using all six microhaplotypes was estimated to be > 99.99%. Estimates of MOI determined naively and using THE REAL McCOIL were comparable, with no significant differences observed (*P* = 0.77, Wilcoxon signed rank test; Supplementary Fig. 8).

**Table 1:** Diversity metrics of six microhaplotype marker in the 20 pre-treatment patient samples using nanopore AmpSeq.

Marker	Size (bp)	Number of haplotypes	Number of SNPs	Expected heterozygosity (H_E_)
*ama1*	201	22	22	0.97
*celtos*	123	21	13	0.96
*cpmp*	192	28	39	0.99
*cpp*	184	19	24	0.95
*csp*	143	17	13	0.95
*surfin1.1*	175	14	15	0.91

Direct haplotype comparison for markers *cpmp*, *cpp* and *csp* between nanopore and Illumina sequencing was possible as the shorter nanopore amplicons were nested within the amplicons previously sequenced by Illumina in the same 20 paired patient samples (*n* = 40)^13^. Corresponding regions from Illumina data were extracted to determine the proportion of haplotypes identified by Illumina that were also detected using nanopore sequencing. Haplotype concordance between the two platforms was high: 100% (30/30) for *cpmp*, 100% (19/19) for *cpp*, and 95% (19/20) for *csp* (Supplementary Fig. 9a-c).

### Distinguishing recrudescence from new infection in an antimalarial drug trial

The ability of nanopore AmpSeq to reliably distinguish recrudescence from new infection was assessed using 20 paired patient samples. Concordance across the six microhaplotype markers was high, with all markers providing the same outcome for 85% (17/20) of patients (Table 2). Fleiss’ Kappa indicated almost perfect agreement (κ = 0.825, z = 13.9, *p* < 0.001). Comparisons with AmpSeq data from Illumina sequencing^13^ yielded nearly identical results (Table 2). One patient sample pair (P09) failed to amplify marker *cpp*, likely due to mutations in the primer binding site, which was also observed previously using Illumina sequencing^13^. The final genotyping outcome was determined using two algorithms: the WHO algorithm (i.e., all markers show recrudescence) and the two out of three algorithm (i.e., ≥ 4/6 markers show recrudescence) (Table 3). Both algorithms yielded concordant results for all 20 patient samples, with 100% agreement with Illumina AmpSeq genotyping outcomes. To further confirm these results, we estimated pairwise genetic relatedness for each of the 20 paired patient samples (baseline and recurrent) using an IBD-based approach^21^. As expected, the four samples classified as new infections showed low to no genetic relatedness (arbitrary IBD threshold < 0.25), while the 16 samples classified as recrudescence showed clonal relatedness (IBD = 1; Table 3, Supplementary Table 12).

**Table 2:** New infection and recrudescence outcome of AmpSeq using illumina and nanopore sequencing using 20 paired pre- and post-treatment samples.

Sample ID	AmpSeq (Illumina) ^13^					AmpSeq (Nanopore)					
	*ama1-D3*	*cpmp*	*cpp*	*csp*	*msp7*	*ama1*	*celtos*	*cpmp*	*cpp*	*csp*	*surfin1.1*
P01	*R*	*R*	*R*	*R*	*R*	*R*	*R*	*R*	*R*	*R*	*R*
P02	*R*	*R*	*R*	*R*	*R*	*R*	*R*	*R*	*R*	*R*	*R*
P03	*R*	*R*	*R*	*R*	*R*	*R*	*R*	*R*	*R*	*R*	*R*
P04	*R*	*R*	*R*	*R*	*R*	*R*	*R*	*R*	*R*	*R*	*R*
P05	*R*	*R*	*R*	*R*	*R*	*R*	*R*	*R*	*R*	*R*	*R*
P06	**NI**	**NI**	**NI**	**NI**	**NI**	**NI**	R	**NI**	**NI**	**NI**	**NI**
P07	*R*	*R*	*R*	*R*	*R*	*R*	*R*	*R*	*R*	*R*	*R*
P08	*R*	*R*	*R*	*R*	*R*	*R*	*R*	*R*	*R*	*R*	*R*
P09	*R*	*R*	ND	*R*	*R*	*R*	*R*	*R*	ND	*R*	*R*
P10	*R*	*R*	*R*	*R*	*R*	*R*	*R*	*R*	*R*	*R*	*R*
P11	*R*	*R*	*R*	*R*	*R*	*R*	*R*	*R*	*R*	*R*	*R*
P12	*R*	*R*	*R*	*R*	*R*	*R*	*R*	*R*	*R*	*R*	*R*
P13	*R*	*R*	*R*	*R*	*R*	*R*	*R*	*R*	*R*	*R*	*R*
P14	*R*	*R*	*R*	*R*	*R*	*R*	*R*	*R*	*R*	*R*	*R*
P15	**NI**	**NI**	**NI**	**NI**	**NI**	**NI**	**NI**	**NI**	*R*	**NI**	**NI**
P16	*R*	*R*	*R*	*R*	*R*	*R*	*R*	*R*	*R*	*R*	*R*
P17	*R*	*R*	*R*	*R*	*R*	*R*	*R*	*R*	*R*	*R*	*R*
P18	**NI**	**NI**	**NI**	**NI**	**NI**	**NI**	*R*	**NI**	**NI**	**NI**	**NI**
P19	*R*	*R*	*R*	*R*	*R*	*R*	*R*	*R*	*R*	*R*	*R*
P20	**NI**	**NI**	**NI**	**NI**	*R*	**NI**	**NI**	**NI**	**NI**	**NI**	**NI**

Patient samples are listed by patient identification number (e.g., P01). Patient samples shaded in grey represent recurrent samples with the *P. falciparum* *ATP4* G538S mutation associated with resistance to cipargamin. R = recrudescence outcome (Italics). NI = new infection outcome (Bold). ND = not determined (no sequencing result). Illumina data were previously generated^13^.

**Table 3:** Results of molecular correction outcomes determined by AmpSeq using illumina and nanopore of 20 paired pre- and post-treatment samples.

Sample ID	AmpSeq Illumina (*ama1-D3, cpmp, cpp, csp, msp7*) ^13^		AmpSeq Nanopore (*ama1, celtos, cpmp, cpp, csp, surfin1.1*)		
	WHO algorithm	Two out of three algorithm	WHO algorithm	Two out of three algorithm	Pairwise IBD (95%CI)
P01	*R*	*R*	*R*	*R*	*1 (0.71–1.00)*
P02	*R*	*R*	*R*	*R*	*1 (0.65–1.00)*
P03	*R*	*R*	*R*	*R*	*1 (0.68–1.00)*
P04	*R*	*R*	*R*	*R*	*1 (0.57–1.00)*
P05	*R*	*R*	*R*	*R*	*1 (0.71–1.00)*
P06	**NI**	**NI**	**NI**	**NI**	**0.11 (0.00–0.52)**
P07	*R*	*R*	*R*	*R*	*1 (0.71–1.00)*
P08	*R*	*R*	*R*	*R*	*1 (0.69–1.00)*
P09	*R*	*R*	*R*	*R*	*1 (0.65–1.00)*
P10	*R*	*R*	*R*	*R*	*1 (0.71–1.00)*
P11	*R*	*R*	*R*	*R*	*1 (0.71–1.00)*
P12	*R*	*R*	*R*	*R*	*1 (0.63–1.00)*
P13	*R*	*R*	*R*	*R*	*1 (0.69–1.00)*
P14	*R*	*R*	*R*	*R*	*1 (0.63–1.00)*
P15	**NI**	**NI**	**NI**	**NI**	**0.1 (0.00–0.52)**
P16	*R*	*R*	*R*	*R*	*1 (0.69–1.00)*
P17	*R*	*R*	*R*	*R*	*1 (0.71–1.00)*
P18	**NI**	**NI**	**NI**	**NI**	**0 (0.00–0.37)**
P19	*R*	*R*	*R*	*R*	*1 (0.68–1.00)*
P20	**NI**	**NI**	**NI**	**NI**	**0 (0.00–0.27)**
Total number of recrudescence’s	16	16	16	16	16

Patient samples are listed by patient identification number (e.g., P01). Patient samples shaded in grey represent recurrent samples with the *P. falciparum* *ATP4* G538S mutation associated with resistance to cipargamin. Both, the WHO and two out of three algorithms (≥ 4/5 for Illumina and ≥ 4/6 for nanopore) were used. In addition, the results by genetic relatedness (i.e., IBD) are shown for results by nanopore AmpSeq. R = recrudescence outcome (Italics). NI = new infection outcome (Bold). Illumina data were previously generated^13^.

## Discussion

Molecular correction is critical for accurate estimation of antimalarial drug efficacy. Genotyping assays must be sensitive enough to detect minority clones present at low frequency in polyclonal infections, reproducible, and use markers with high diversity^9^. Previous studies have shown that AmpSeq provides superior results in distinguishing recrudescence from new infections compared to other genotyping methods, reinforcing its potential as the standard for molecular correction^12,13,20^. However, many smaller laboratories in malaria-endemic countries still rely on shipping samples internationally for sequencing. Although capacity is gradually improving, efficient, cost-effective, reliable and accessible tools are needed to strengthen the genomic capacity of research and public health institutions in malaria-endemic countries before AmpSeq can be adopted for routine TES.

In this study, we optimized a multiplexed nanopore AmpSeq panel for *P. falciparum* microhaplotypes. Our panel accurately detects minority clones in polyclonal infections and is thus a promising tool for distinguishing recrudescence from new infection in clinical trials. The assay achieved high and uniform coverage across all targeted microhaplotypes - critical for accurate variant calling, particularly when identifying minority alleles. The method proved highly sensitive, specific and reproducible, detecting minor alleles (present at 10 parasites/µL) at WSAF as low as 0.3% (i.e., 1:100:100:100). This sensitivity is comparable to that of *P. falciparum* AmpSeq panels developed for Illumina platforms^14–16^. Assay performance was robust, with high intra- and inter-assay reproducibility. To ensure high specificity while maintaining sensitivity, we excluded reads with < 99% accuracy (Q < 20) and applied a WSAF threshold of ≥ 0.1%, resulting in only one false-positive haplotype.

While our study and others^11,35^ have shown that a WSAF threshold as low as ≥ 0.1% enables robust detection of minority clones, the optimal cutoff may vary depending on the study context. A stringent cutoff is required for excluding sequencing artefacts, whereas a less stringent cutoff increases sensitivity to detect minority clones. In regulatory clinical trials, where robustness and reproducibility are paramount, a conservative cutoff that errs on the side of overestimating treatment failure may be preferable. Importantly, cutoff selection should be guided by appropriate internal controls within each sequencing experiment. In our study, the chosen threshold was supported by results from control mixtures of *P. falciparum* strains, which defined the technical detection limit for minority clones. Several published *P. falciparum* AmpSeq studies have adopted WSAF cutoffs of ~ 1–2%, striking an effective balance between minimizing false positives and retaining sensitivity^12,14–16,18,19,41^. The assay was effective on strain mixtures with parasitemias as low as 40 parasites/µL and maintained high coverage in paired patient samples, including nine with low parasitemia levels below 500 parasites/µL (31–462 parasites/µL). In previous work, we also demonstrated that the assay performs robustly on dried blood spots with parasitemias as low as 250 parasites/µL^26^.

The entire workflow - from DNA extraction to results - can be completed in approximately three to four days. Estimated running costs are approximately US$25 per sample when multiplexing 96 samples (Supplementary Table 13). Further cost reductions may be possible with higher degrees of multiplexing as the number of barcodes increases and through flow cell reuse following washing. In addition to the 96 native barcodes currently available, custom barcodes could be added to the amplicons to generate dual-barcoded libraries, thereby enabling multiplexing of > 96 samples per flow cell.

To fully leverage the information content from diverse microhaplotype loci, particularly in polyclonal infections, it is critical to use haplotype inference tools capable of accurately identifying complex infections with unknown haplotype proportions. While such tools exist for short-read Illumina data, only our previously developed workflow effectively captures all information from polyclonal infections using nanopore data^26^. We adapted existing short-read haplotype inference tools, HaplotypR^11^ and DADA2 ^38^, using only high-quality reads and appropriate cutoffs which are required by DADA2^38^. Given the short amplicon length (~ 200 bp), sequencing errors are minimal, allowing for reliable haplotype inference and distinguishing true variation from errors. However, this approach may not be suitable for longer amplicons (> 1 kb), where error accumulation complicates the differentiation between true variants and sequencing errors. Hence, nanopore AmpSeq of short microhaplotypes is more robust against sequencing errors, compared to the previously utilized longer amplicons^23,24^, while maintaining high discriminatory power.

Our panel demonstrated high genetic diversity (H_E_ > 0.9 for all markers) and low allelic frequencies in patient samples, with up to 28 distinct haplotypes for *cpmp*, providing high discriminatory power. For example, based on the H_E_ of all six microhaplotypes across all 20 pre-treatment samples, there would be a > 99.99% chance of determining that strains are different. MOI estimates in patient samples were highly similar whether based on the locus with the greatest number of alleles or using THE REAL McCOIL. Concordance between markers *cpmp*, *cpp*, and *csp* and Illumina results was very high. However, two of the six markers in our panel - *ama1* (FCB1 and K1) and *csp* (FCB1 and HB3) - were unable to distinguish between two well-characterized laboratory strains. These findings highlight the need of validating new genotyping methods across geographically diverse strains and evaluating marker diversity and allele frequency in TES study site samples, as marker performance may vary substantially between regions.

Previous studies have shown that molecular correction outcomes can vary widely depending on the algorithm used^13,20^. In this study, the six markers consistently yielded concordant results in 85% of the samples analyzed, and more importantly, the final corrected outcomes were 100% identical across all samples, regardless of the algorithm applied. The results were identical to those previously obtained with longer amplicons sequenced on the Illumina platform^13^. The high concordance between the two methods supports multiplexed nanopore AmpSeq as a promising tool for distinguishing different parasite clones. Additionally, using Dcifer, a relatedness-based approach that accounts for population allele frequencies and provides uncertainty measures^21^, yielded the same results as the WHO and two out of three algorithms. Such novel approaches could potentially provide even more robust estimates of interhost relatedness by using a larger set of microhaplotypes and accounting for missing alleles, thus providing a more accurate corrected estimate.

ONT devices provide a cost-effective alternative to traditional sequencing methods, offering rapid data generation and enabling in-country sequencing, thus minimizing result turnaround times. Despite its apparent benefits and continuous improvements in sequencing performance, nanopore sequencing of *P. falciparum* remains underutilized^22–24,26^. Previous malaria studies have demonstrated the feasibility of nanopore AmpSeq for various applications^22–24,26–28^, including sequencing in malaria-endemic countries^23,26^; however, this is the first study to use nanopore sequencing to distinguish recrudescence from new infection in antimalarial drug efficacy studies. Although nanopore sequencing allows long-read AmpSeq, we opted for short amplicons to distinguish recrudescence from new infection for several reasons. While longer microhaplotype reads may offer greater discriminatory power in resolving haplotypes by capturing more SNPs and thus increasing genetic diversity, shorter amplicons generally exhibit higher amplification efficiency and can be designed to target the most informative regions, avoiding error-prone homopolymers or tandem repeats. Indeed, nanopore sequencing struggles to accurately resolve low-complexity regions^24,42^, which are abundant in the *P. falciparum* genome^43^. Previous studies using nanopore AmpSeq did not attempt to infer individual haplotypes or detect minority clones in polyclonal infections. Rather, they focused on the prevalence of drug-resistance mutations^22–24,28^.

Our study has several limitations. First, for two markers, *ama1* and *csp*, the LOD could only be determined for two out of four strains due to identical alleles. Second, we did not assess inter-assay reproducibility across different operators or laboratories. Third, the small number of patient samples, with a high proportion of recrudescence, limits the generalizability of the results. Fourth, while our assay was designed to be user-friendly with high and uniform coverage, it was not a direct adaptation of the previously described Illumina AmpSeq protocol^13^. Rather, we employed a multiplex PCR approach targeting short microhaplotypes with optimized amplification efficiency that does not require a prior pre-amplification step (i.e., selective whole genome amplification). As a result, direct comparisons with the Illumina protocol were limited; only three of our amplicons overlapped with regions sequenced by Illumina, allowing for restricted, locus-specific comparisons. Direct cross-platform comparisons will require the use of an identical AmpSeq panel across platforms in future work. Finally, this study did not evaluate the practical utility or in-country implementation of the assay for use in antimalarial drug trials. However, in a previous pilot study, we have successfully used the microhaplotype nanopore AmpSeq assay outside of an advanced laboratory in a malaria-endemic country^26^.

In conclusion, our study demonstrates the feasibility of using rapid, multiplexed nanopore AmpSeq to genotype *P. falciparum* and distinguish recrudescence from new infection, expanding the current applications of nanopore sequencing in malaria research and molecular surveillance. We show that detecting minority clones in polyclonal infections and characterizing within-host diversity is feasible using nanopore AmpSeq targeting short, highly diverse microhaplotypes. Although further studies from clinical drug trials with larger sample sizes are needed to validate the assay’s ability to distinguish recrudescence from new infection, this work represents an important initial proof-of-principle for this approach. ONT sequencing platforms are affordable, portable, and scalable, making them suitable for resource-limited endemic settings. This technology offers a promising solution to allow for in-country sequencing and rapid corrected estimates of drug failure using AmpSeq.

## Supplementary Information

Below is the link to the electronic supplementary material.

Supplementary Material 1

## Data Availability

Raw sequence data is available for download from NCBI’s Sequence Read Archive (https://www.ncbi.nlm.nih.gov/sra) under the accession PRJNA1188139. Haplotype data can be found at (10.5281/zenodo.14176815) . All other data can be found in the supplementary files. A complete laboratory protocol, including materials and primer sequences, is available online at protocols.io (https://www.protocols.io) as " [Rapid multiplexed nanopore amplicon sequencing of Plasmodium falciparum microhaplotype loci] (https://www.protocols.io/view/rapid-multiplexed-nanopore-amplicon-sequencing-of-81wgbrqnqlpk/v1)"^37^.

## Code availability

All analyses were completed in R version 4.1.2 using published R packages, as described in the methods section. Bioinformatics pipeline and code used for haplotype inference can be found at 10.5281/zenodo.14176815. The source code for software HaplotypR, including a tutorial with example datasets, is available at https://github.com/lerch-a/HaplotypR/releases/tag/v0.5.
