# Supplementary material for: Rapid multiplexed nanopore amplicon sequencing to distinguish Plasmodium falciparum recrudescence from new infection in antimalarial drug trials
Source: Sci Rep. 2025 Oct 22;15:36941. doi: 10.1038/s41598-025-20925-7 (PMC12546925; doi:10.1038/s41598-025-20925-7)
Supplement: Supplementary file 1 — Supplementary Material 1 [file 41598_2025_20925_MOESM1_ESM.docx]

**SUPPLEMENTARY INFORMATION**

Supplementary information for the paper Holzschuh et al. ***Rapid*** ***multiplexed nanopore amplicon sequencing to distinguish recrudescence from new infection in antimalarial drug trials***

TABLE OF CONTENTS

1. Supplementary Table 1 Page 2
2. Supplementary Table 2 Page 3
3. Supplementary Table 3 Page 4
4. Supplementary Table 4 Page 5
5. Supplementary Table 5 Page 6
6. Supplementary Table 6 Page 7
7. Supplementary Table 7 Page 8
8. Supplementary Table 8 Page 9
9. Supplementary Table 9 Page 10
10. Supplementary Table 10 Page 11
11. Supplementary Table 11 Page 14
12. Supplementary Table 12 Page 15
13. Supplementary Table 13 Page 16
14. Supplementary Fig. 1 Page 17
15. Supplementary Fig. 2 Page 18
16. Supplementary Fig. 3 Page 19
17. Supplementary Fig. 4 Page 20
18. Supplementary Fig. 5 Page 21
19. Supplementary Fig. 6 Page 22
20. Supplementary Fig. 7 Page 23
21. Supplementary Fig. 8 Page 24
22. Supplementary Fig. 9 Page 25
23. Supplementary Text Page 26

**Supplementary Table 1. Laboratory strain mixtures at different ratios.** Mixtures were prepared and used previously ^2^. Total indicates the overall parasites/µL in each strain mixture.

| Sample ID | Strain ratio | | | | Parasites/µL blood | | | | |
| --- | --- | --- | --- | --- | --- | --- | --- | --- | --- |
|  | **3D7** | **K1** | **HB3** | **FCB1** | **3D7** | **K1** | **HB3** | **FCB1** | **Total** |
| S01_new | 1 | 0 | 0 | 0 | 1,000 | 0 | 0 | 0 | 1,000 |
| S02_new | 0 | 1 | 0 | 0 | 0 | 1,000 | 0 | 0 | 1,000 |
| S03_new | 0 | 0 | 1 | 0 | 0 | 0 | 1,000 | 0 | 1,000 |
| S04_new | 0 | 0 | 0 | 1 | 0 | 0 | 0 | 1,000 | 1,000 |
| S05_new | 1 | 1 | 1 | 1 | 1,000 | 1,000 | 1,000 | 1,000 | 4,000 |
| S05 | 1 | 1 | 1 | 1 | 10 | 10 | 10 | 10 | 40 |
| S06 | 5 | 1 | 1 | 1 | 50 | 10 | 10 | 10 | 80 |
| S07 | 1 | 5 | 1 | 5 | 10 | 50 | 10 | 50 | 120 |
| S08 | 1 | 5 | 5 | 5 | 10 | 50 | 50 | 50 | 160 |
| S09 | 1 | 10 | 1 | 1 | 10 | 100 | 10 | 10 | 130 |
| S10 | 1 | 1 | 10 | 10 | 10 | 10 | 100 | 100 | 220 |
| S11 | 10 | 1 | 10 | 10 | 100 | 10 | 100 | 100 | 310 |
| S12 | 1 | 1 | 15 | 1 | 10 | 10 | 150 | 10 | 180 |
| S13 | 15 | 15 | 1 | 1 | 150 | 150 | 10 | 10 | 320 |
| S14 | 15 | 15 | 1 | 15 | 150 | 150 | 10 | 150 | 460 |
| S15 | 1 | 1 | 1 | 20 | 10 | 10 | 10 | 200 | 230 |
| S16 | 20 | 1 | 20 | 1 | 200 | 10 | 200 | 10 | 420 |
| S17 | 20 | 20 | 20 | 1 | 200 | 200 | 200 | 10 | 610 |
| S18 | 50 | 1 | 1 | 1 | 500 | 10 | 10 | 10 | 530 |
| S19 | 1 | 50 | 1 | 50 | 10 | 500 | 10 | 500 | 1,020 |
| S20 | 1 | 50 | 50 | 50 | 10 | 500 | 500 | 500 | 1,510 |
| S21 | 100 | 1 | 1 | 1 | 1,000 | 10 | 10 | 10 | 1,030 |
| S22 | 1 | 100 | 1 | 100 | 10 | 1,000 | 10 | 1,000 | 2,020 |
| S23 | 1 | 100 | 100 | 100 | 10 | 1,000 | 1,000 | 1,000 | 3,010 |

**Supplementary Table 2. List of Independent Ethics Committees (IECs) or Institutional Review Boards (IRBs) by study center.**

| Center No. | Ethics Committee or Institutional Review Board | City, State/Province, Postal Code  Country |
| --- | --- | --- |
| 1001 | Comite d'ethique de la FMPOS (The Ethics Committee of the FMPOS) | Bamako  Mali |
| 1002 | Comite d'ethique de la FMPOS (The Ethics Committee of the FMPOS) | Bamako  Mali |
| 3001 | Mbarara University of Research and Technology Research Ethics Committee | Mbarara  Uganda |
| 3002 | Uganda National Council for Science and Technology (UNCST) | Uganda |
| 3003 | Makerere University | Kampala  Uganda |
| 4001 | Navrongo Health Research Centre | Navrongo  Ghana |
| 4002 | Kintampo Health Research Centre  Institutional Ethics Committee | Kintampo  Ghana |
| 5001 | Comite d'Ethique Institutionnel Centre de Recherches Medicales de Lambarene  Foundation Internationale deI'Hopital alber Schweitzer (Site IRB)  Comite' National d'Ethique de la recherche Au Gabon (CNER) (Nationla IRB) | Lambarene  Libreville  Gabon |
| 6001 | Rwanda National Ethics Committee (RNEC) | Kigali  Rwanda |
| 6002 | Rwanda National Ethics Committee (RNEC) | Kigali  Rwanda |

**Supplementary Table 3.** **Parasitemia of the 20 paired patient samples.** Parasite counts (Giemsa-stained thick and thin films) were made per 200 white blood cells (or if the count was <100 parasites, counting was continued for up to 500 white blood cells).

| Sample ID | Parasitemia (parasites/μL) |
| --- | --- |
| P01_D0 | 12,080 |
| P01_DX | 2,877 |
| P02_D0 | 6,680 |
| P02_DX | 36 |
| P03_D0 | 6,994 |
| P03_DX | 462 |
| P04_D0 | 14,593 |
| P04_DX | 228 |
| P05_D0 | 1,966 |
| P05_DX | 40 |
| P06_D0 | 6,903 |
| P06_DX | 557 |
| P07_D0 | 10,755 |
| P07_DX | 3,291 |
| P08_D0 | 5,047 |
| P08_DX | 9,091 |
| P09_D0 | 33,930 |
| P09_DX | 11,163 |
| P10_D0 | 3,838 |
| P10_DX | 13,270 |
| P11_D0 | 28,805 |
| P11_DX | 5,763 |
| P12_D0 | 27,735 |
| P12_DX | 278 |
| P13_D0 | 22,724 |
| P13_DX | 931 |
| P14_D0 | 15,847 |
| P14_DX | 154 |
| P15_D0 | 8,527 |
| P15_DX | 31 |
| P16_D0 | 18,624 |
| P16_DX | 257 |
| P17_D0 | 15,546 |
| P17_DX | 16,885 |
| P18_D0 | 8,256 |
| P18_DX | 2,025 |
| P19_D0 | 10,393 |
| P19_DX | 52 |
| P20_D0 | 33,143 |
| P20_DX | 9,191 |

**Supplementary Table 4. P. falciparum highly polymorphic microhaplotypes targeted by the 6-plex amplicon panel.** Median length across all amplicons is 231 bp (range: 179-250).

| Gene name (*PlasmoDB* Gene ID) | Chromosome | Start | End | Amplicon size (including primer) | Associated phenotype |
| --- | --- | --- | --- | --- | --- |
| Apical membrane antigen 1, *ama1* (PF3D7_1133400) | Pf3D7_11_v3 | 1294271 | 1294520 | 250 bp | Vaccine candidate antigen; potential for use as a marker of complexity of infection |
| Cell traversal protein for ookinetes and sporozoites, *celtos*  (PF3D7_1216600) | Pf3D7_12_v3 | 659859 | 660037 | 179 bp | Vaccine candidate antigen, potential for use as a marker of complexity of infection |
| Conserved Plasmodium membrane protein, *cpmp* (PF3D7_0104100) | Pf3D7_01_v3 | 180130 | 180370 | 241 bp | Potential for use as a marker of complexity of infection |
| Conserved Plasmodium protein, *cpp* (PF3D7_1475800) | Pf3D7_14_v3 | 3121036 | 3121267 | 232 bp | Potential for use as a marker of complexity of infection |
| Circumsporozoite protein, *csp* (PF3D7_0304600) | Pf3D7_03_v3 | 221468 | 221655 | 188 bp | Leading vaccine and monoclonal antibody target antigen; potential for use as a marker of complexity of infection |
| Surface-associated interspersed protein 1.1, *surfin1.1* (PF3D7_0113100) | Pf3D7_01_v3 | 495942 | 496171 | 230 bp | Potential for use as a marker of complexity of infection |

**Supplementary Table 5. Primer sequences for the 6-plex microhaplotype panel.**

| Primer | Direction | Sequence | Source | Stock concentration (mM) | Concentration in final pool (µM) | Final concentration in PCR reaction (µM) |
| --- | --- | --- | --- | --- | --- | --- |
| *ama1* | Forward | GAACTCAATATAGACTTCCATCAGG | [2] | 1 | 7 | 0.14 |
|  | Reverse | CCTGCATGTCTTGAACATAAAGTC | [2] | 1 | 7 | 0.14 |
| *celtos* | Forward | CTGGTACTATTATACCATATGTTGC | [2] (adapted from [3]) | 1 | 15 | 0.3 |
|  | Reverse | TCACCAACCTTTTTAGAATCAAGC | [2] (adapted from [3]) | 1 | 15 | 0.3 |
| *cpmp* | Forward | GGAAGCTATAGGTATCAGATCC | [2] | 1 | 10 | 0.2 |
|  | Reverse | TAGAATACGTGCTTTATAAACAAAGAG | [2] | 1 | 10 | 0.2 |
| *cpp* | Forward | AACACAATCTTCCTTAGCCAATTC | [2] | 1 | 8 | 0.16 |
|  | Reverse | ATTACTACCTTTCAGCATATCCGA | [2] | 1 | 8 | 0.16 |
| *csp* | Forward | GACCCAAACCGAAATGTAGATG | [2] | 1 | 10 | 0.2 |
|  | Reverse | GAGCCAGGCTTTATTCTAACTTG | [2] | 1 | 10 | 0.2 |
| *surfin1.1* | Forward | CACCAAAATATTATATACCACAAGAC | [2] (adapted from [3]) | 1 | 15 | 0.3 |
|  | Reverse | GGAAAATCTTTGGTGGGAAAAATAG | [2] (adapted from [3]) | 1 | 15 | 0.3 |

**Supplementary Table 6. Reaction mixture of 6-plex microhaplotype panel.**

| Reagent | Conc. | Final conc. | 1X for 25 μL |
| --- | --- | --- | --- |
|  |  |  |  |
| ddH2O |  |  | 8 μL |
| 2X KAPA HiFi HotStart ReadyMix | 2X | 1X | 12.5 μL |
| Primer mix (fw & rv) | 7-15µM | 140-300nM | 0.5 μL |
| Template (gDNA) |  |  | 4 μL |

**Supplementary Table 7. Thermal cycling conditions of 6-plex microhaplotype panel.**

| Cycling step | Temp (ºC) | Time | # of cycles |
| --- | --- | --- | --- |
|  |  |  |  |
| Initial denaturation | 95 | 3 min | 1 |
| Denaturation | 98 | 15 sec | 35 |
| Annealing | 56 | 15 sec |  |
| Extension | 72 | 30 sec |  |
| Final Extension | 72 | 2 min | 1 |
| Hold | 4 | ∞ |  |

**Supplementary Table 8. ONT sequencing run characteristics.**

| Run | ONT chemistry used | Run time | Samples per run (of which controls) | Total reads | Mean  Q-score (accuracy)* | Median  Q-score (accuracy)* | Reads with ≥Q20, pass (%) |
| --- | --- | --- | --- | --- | --- | --- | --- |
| Run 1 (Control mixtures) | Kit 14, R10.4.1 | 23h 30min | 75 (3) | 11,151,532 | 16.2 (97.6%) | 21.6 (99.3%) | 6,882,758 (61.7%) |
| Run 2 (Control mixtures) | Kit 14, R10.4.1 | 20h40min | 75 (3) | 11,217,189 | 13.7 (95.7%) | 19.2 (98.8%) | 4,976,633 (44.4%) |
| Run 3 (Paired patient samples) | Kit 14, R10.4.1 | 18h 10min | 44 (4) | 6,510,890 | 15.6 (97.2%) | 20.8 (99.2%) | 3,661,566 (56.2%) |

* With *dorado v0.8.2* using the super-accurate (sup) model (dna_r10.4.1_e8.2_400bps_sup@v5.0.0).

**Supplementary Table 9. Different primer concentrations tested for primer balancing.** Primer pools ‘old’, ‘new1, ‘new2, and ‘new3’ were tested at annealing temperatures 56 ºC and 58 ºC. 56 ºC was found to be superior and ultimately in a second sequencing round, primer pool ‘new4’ (highlighted) was found to produce most even coverage across all amplicons.

| Amplicon | Primer concentrations in different pools (µM) | | | | | |
| --- | --- | --- | --- | --- | --- | --- |
|  | old* | new1 | new2 | new3 | **new4** | new5 |
| *ama1* | 5 | 15 | 15 | 10 | **7** | 8 |
| *celtos* | 10 | 10 | 10 | 10 | **15** | 12 |
| *cpmp* | 10 | 15 | 10 | 10 | **10** | 10 |
| *cpp* | 5 | 20 | 15 | 10 | **8** | 9 |
| *csp* | 5 | 5 | 5 | 10 | **10** | 10 |
| *surfin1.1* | 15 | 10 | 10 | 10 | **15** | 12 |

*Primer pool from ^2^.

**Supplementary Table 10.** **LOD for minority clones in control mixtures between the two MinION sequencing runs.** Each cell shows how many of the triplicates detected the minority clones at a WSAF threshold of ≥0.1%. For marker *ama1* and *csp*, minority clone detection could only be done for two strains (3D7 and HB3 for *ama1*; 3D7 and K1 for *csp*) when one strain was in majority and the other in minority, thus some ratios are coded as “NA”. The ratios represent the ratio of the four Plasmodium falciparum laboratory strains assessed in this study: 3D7:K1:HB3:FCB1, with the concentration of the minority clone always being 10 parasites/μL. The asterisk (*) indicates the detection of one false-positive haplotype in one replicate at 1.02% WSAF. NA: not applicable.

| Sample ID | Strain ratio | *ama1* | | *celtos* | | *cpmp* | | *cpp* | | *csp* | | *surfin1.1* | |
| --- | --- | --- | --- | --- | --- | --- | --- | --- | --- | --- | --- | --- | --- |
|  |  | **Run1** | **Run2** | **Run1** | **Run2** | **Run1** | **Run2** | **Run1** | **Run2** | **Run1** | **Run2** | **Run1** | **Run2** |
| S05 | 1:1:1:1 (1:1) | NA | NA | 3/3 | 3/3 | 3/3 | 3/3 | 3/3 | 3/3 | NA | NA | 3/3 | 3/3 |
| S06 | 5:1:1:1 (1:5) | 3/3 | 3/3 | 3/3 | 3/3 | 3/3 | 3/3 | 3/3 | 3/3 | 3/3 | 3/3 | 3/3 | 3/3 |
| S07 | 1:5:1:5 (1:5) | NA | NA | 3/3 | 3/3 | 3/3 | 3/3 | 3/3 | 3/3 | 3/3 | 3/3 | 3/3 | 3/3 |
| S08 | 1:5:5:5 (1:5) | 3/3 | 3/3 | 3/3 | 3/3 | 3/3 | 3/3 | 3/3 | 3/3 | 3/3 | 3/3 | 3/3 | 3/3 |
| S09 | 1:10:1:1 (1:10) | NA | NA | 3/3 | 3/3 | 3/3 | 3/3 | 3/3 | 3/3 | 3/3 | 3/3 | 3/3 | 3/3 |
| S10 | 1:1:10:10 (1:10) | 3/3 | 3/3 | 3/3 | 3/3 | 3/3 | 3/3 | 3/3 | 3/3 | NA | NA | 3/3 | 3/3 |
| S11 | 10:1:10:10 (1:10) | NA | NA | 3/3 | 3/3 | 3/3 | 3/3 | 3/3 | 3/3 | 3/3 | 3/3 | 3/3* | 3/3 |
| S12 | 1:1:15:1 (1:15) | 3/3 | 3/3 | 3/3 | 3/3 | 3/3 | 3/3 | 3/3 | 3/3 | NA | NA | 3/3 | 3/3 |
| S13 | 15:15:1:1 (1:15) | 3/3 | 3/3 | 3/3 | 3/3 | 3/3 | 3/3 | 3/3 | 3/3 | NA | NA | 3/3 | 3/3 |
| S14 | 15:15:1:15 (1:15) | 3/3 | 3/3 | 3/3 | 3/3 | 3/3 | 3/3 | 3/3 | 3/3 | NA | NA | 3/3 | 3/3 |
| S15 | 1:1:1:20 (1:20) | NA | NA | 3/3 | 3/3 | 3/3 | 3/3 | 3/3 | 3/3 | NA | NA | 3/3 | 3/3 |
| S16 | 20:1:20:1 (1:20) | NA | NA | 3/3 | 3/3 | 3/3 | 3/3 | 3/3 | 3/3 | 3/3 | 3/3 | 3/3 | 3/3 |
| S17 | 20:20:20:1 (1:20) | NA | NA | 3/3 | 3/3 | 3/3 | 3/3 | 3/3 | 3/3 | NA | NA | 3/3 | 3/3 |
| S18 | 50:1:1:1 (1:50) | 3/3 | 3/3 | 3/3 | 3/3 | 3/3 | 3/3 | 3/3 | 3/3 | 3/3 | 3/3 | 3/3 | 3/3 |
| S19 | 1:50:1:50 (1:50) | NA | NA | 3/3 | 3/3 | 3/3 | 3/3 | 3/3 | 3/3 | 3/3 | 3/3 | 3/3 | 3/3 |
| S20 | 1:50:50:50 (1:50) | 3/3 | 3/3 | 3/3 | 3/3 | 3/3 | 3/3 | 3/3 | 3/3 | 3/3 | 3/3 | 3/3 | 3/3 |
| S21 | 100:1:1:1 (1:100) | 3/3 | 3/3 | 3/3 | 3/3 | 3/3 | 3/3 | 3/3 | 3/3 | 3/3 | 3/3 | 3/3 | 3/3 |
| S22 | 1:100:1:100 (1:100) | NA | NA | 3/3 | 3/3 | 2/3 | 1/3 | 3/3 | 1/3 | 3/3 | 3/3 | 3/3 | 3/3 |
| S23 | 1:100:100:100 (1:100) | 3/3 | 3/3 | 3/3 | 3/3 | 3/3 | 3/3 | 3/3 | 3/3 | 3/3 | 3/3 | 3/3 | 3/3 |

**Supplementary Table 11. SNP position in the six microhaplotype markers found in the 20 pre-treatment samples.** REF refers to strain 3D7.

| Marker | Position in amplicon | REF | ALT |
| --- | --- | --- | --- |
| *ama1* | 46 | T | A |
| *ama1* | 60 | C | A |
| *ama1* | 75 | G | A |
| *ama1* | 79 | T | A |
| *ama1* | 83 | T | G |
| *ama1* | 119 | G | A |
| *ama1* | 121 | A | T |
| *ama1* | 126 | T | A/C |
| *ama1* | 130 | G | A |
| *ama1* | 146 | G | A |
| *ama1* | 149 | G | C |
| *ama1* | 150 | A | G |
| *ama1* | 151 | A | T |
| *ama1* | 156 | G | A |
| *ama1* | 158 | C | G |
| *ama1* | 159 | A | T/G |
| *ama1* | 161 | T | C |
| *ama1* | 163 | T | G/A |
| *ama1* | 170 | G | A |
| *ama1* | 172 | T | A |
| *ama1* | 176 | A | G |
| *ama1* | 179 | T | G |
| *celtos* | 29 | T | G |
| *celtos* | 30 | C | G |
| *celtos* | 36 | C | T |
| *celtos* | 39 | G | C |
| *celtos* | 40 | A | T |
| *celtos* | 42 | T | G |
| *celtos* | 48 | A | C |
| *celtos* | 51 | A | G |
| *celtos* | 63 | C | A/T |
| *celtos* | 86 | G | T/A |
| *celtos* | 87 | A | G/T |
| *celtos* | 97 | T | G |
| *celtos* | 98 | T | C |
| *cpmp* | 7 | A | G |
| *cpmp* | 8 | C | A |
| *cpmp* | 9 | T | A |
| *cpmp* | 10 | A | G |
| *cpmp* | 11 | C | A |
| *cpmp* | 19 | A | C |
| *cpmp* | 21 | A | C |
| *cpmp* | 23 | T | G |
| *cpmp* | 24 | T | G |
| *cpmp* | 28 | T | C |
| *cpmp* | 30 | T | C |
| *cpmp* | 32 | C | A |
| *cpmp* | 34 | G | A |
| *cpmp* | 35 | A | G |
| *cpmp* | 41 | A | T |
| *cpmp* | 65 | T | A |
| *cpmp* | 66 | C | T |
| *cpmp* | 79 | G | C |
| *cpmp* | 82 | A | G |
| *cpmp* | 113 | A | C |
| *cpmp* | 119 | C | A |
| *cpmp* | 126 | G | A |
| *cpmp* | 134 | G | T |
| *cpmp* | 137 | A | C |
| *cpmp* | 142 | C | T |
| *cpmp* | 146 | A | G |
| *cpmp* | 148 | G | C |
| *cpmp* | 149 | G | A/T |
| *cpmp* | 151 | A | T |
| *cpmp* | 153 | G | A |
| *cpmp* | 154 | G | A |
| *cpmp* | 155 | A | G/T |
| *cpmp* | 158 | A | C |
| *cpmp* | 160 | C | A |
| *cpmp* | 163 | C | A |
| *cpmp* | 166 | A | G |
| *cpmp* | 177 | G | A |
| *cpmp* | 182 | C | A/T |
| *cpmp* | 190 | C | G |
| *cpp* | 17 | A | G |
| *cpp* | 32 | G | C/A |
| *cpp* | 33 | G | A |
| *cpp* | 34 | T | A |
| *cpp* | 36 | C | T |
| *cpp* | 40 | G | T |
| *cpp* | 42 | G | A |
| *cpp* | 45 | G | A |
| *cpp* | 53 | T | A |
| *cpp* | 54 | C | T |
| *cpp* | 61 | A | G/C |
| *cpp* | 62 | A | G |
| *cpp* | 65 | G | C |
| *cpp* | 68 | C | T |
| *cpp* | 70 | T | A |
| *cpp* | 74 | T | C |
| *cpp* | 77 | G | T/C |
| *cpp* | 80 | C | G |
| *cpp* | 82 | G | T |
| *cpp* | 86 | T | C |
| *cpp* | 89 | A | C |
| *cpp* | 90 | C | A |
| *cpp* | 164 | G | C |
| *cpp* | 168 | G | A |
| *csp* | 3 | A | G |
| *csp* | 11 | T | A |
| *csp* | 19 | G | A |
| *csp* | 57 | A | C |
| *csp* | 66 | A | G |
| *csp* | 67 | A | C |
| *csp* | 69 | G | A/C |
| *csp* | 78 | A | C |
| *csp* | 80 | C | G |
| *csp* | 82 | A | C/T/G |
| *csp* | 87 | C | A |
| *csp* | 88 | A | G |
| *csp* | 96 | C | A |
| *surfin1.1* | 13 | G | A |
| *surfin1.1* | 25 | G | C |
| *surfin1.1* | 30 | T | C |
| *surfin1.1* | 37 | A | G |
| *surfin1.1* | 69 | G | C/T |
| *surfin1.1* | 74 | A | C |
| *surfin1.1* | 100 | G | C |
| *surfin1.1* | 105 | A | G |
| *surfin1.1* | 114 | G | A |
| *surfin1.1* | 116 | G | A |
| *surfin1.1* | 117 | C | T |
| *surfin1.1* | 137 | C | T |
| *surfin1.1* | 151 | C | A |
| *surfin1.1* | 174 | A | T |
| *surfin1.1* | 175 | T | C |

**Supplementary Table 12. Pairwise IBD for the 20 paired patient samples using nanopore AmpSeq.** IBD was estimated using haplotypes from the six microhaplotype nanopore AmpSeq data. Pairwise P values were determined by likelihood-ratio adjusted for 1-sided tests and 95% CI for relatedness estimates were produced using the likelihood ratio both implemented in Dcifer ^4^. Green shading indicates new infection outcome, red shading indicates recrudescence outcome.

| Sample ID | Pairwise IBD (95%CI) | *P* value |
| --- | --- | --- |
| P01 | 1 (0.71 – 1.00) | 2.20e-09 |
| P02 | 1 (0.65 – 1.00) | 2.79e-06 |
| P03 | 1 (0.68 – 1.00) | 3.31e-07 |
| P04 | 1 (0.57 – 1.00) | 0.00015 |
| P05 | 1 (0.71 – 1.00) | 5.92e-10 |
| P06 | 0.112 (0.00 – 0.52) | >0.05 |
| P07 | 1 (0.71 – 1.00) | 2.94e-09 |
| P08 | 1 (0.69 – 1.00) | 5.83e-08 |
| P09 | 1 (0.65 – 1.00) | 1.52e-07 |
| P10 | 1 (0.71 – 1.00) | 3.11e-09 |
| P11 | 1 (0.71 – 1.00) | 2.48e-10 |
| P12 | 1 (0.63 – 1.00) | 9.66e-06 |
| P13 | 1 (0.69 – 1.00) | 7.77e-08 |
| P14 | 1 (0.63 – 1.00) | 1.53e-05 |
| P15 | 0.1 (0.00 – 0.52) | >0.05 |
| P16 | 1 (0.69 – 1.00) | 8.41e-08 |
| P17 | 1 (0.71 – 1.00) | 8.93e-10 |
| P18 | 0 (0.00 – 0.37) | >0.05 |
| P19 | 1 (0.68 – 1.00) | 2.60e-07 |
| P20 | 0 (0.00 – 0.27) | >0.05 |

**Supplementary Table 12. Consumable costs for nanopore AmpSeq on a standard MinION Flow Cell (FLO-MIN114).** Number of samples that can be processed for the kit specified are indicated. For DNA extraction, a standard commercial kit was used to estimate costs. The remaining USD $1 per sample absorbs the cost of plasticware, nuclease-free water, ethanol, etc. Costs were retrieved from supplier websites on 2025/08/20.

| Step(s) | Item | Supplier | Item code | Number of samples per unit | Cost per Item (USD) | Cost per sample (USD) |
| --- | --- | --- | --- | --- | --- | --- |
| DNA extraction | QIAamp DNA Micro Kit (50) | Qiagen | 56304 | 50 | $338.00 | $6.76 |
| Multiplex PCR | Primers | Eurofins |  |  |  | $0.01 |
| Multiplex PCR | KAPA HiFi HotStart ReadyMix | Roche | KK2602 | 500 | $500.00 | $1.00 |
| End-prep | NEBNext Ultra II End repair/dA-tailing Module | NEB | E7546L | 384 | $1,200.00 | $3.13 |
| Barcoding | Native Barcoding Kit 96 V14 | ONT | SQK-NBD114.96 | 576 | $900.00 | $1.56 |
| Barcoding | NEB Blunt/TA Ligase Master Mix | NEB | M0367L | 250 | $600.00 | $2.40 |
| Barcoding | AMPure XP beads | Beckman Coulter | A63881 | ~2500 | $2,500.00 | $1.00 |
| Barcoding/Adapter Ligation | Qubit™ 1X dsDNA High Sensitivity (HS) Assay Kit | Thermo Fisher | Q33231 | 500 | $470.00 | $0.94 |
| Adapter Ligation | NEBNext Quick Ligation Module | NEB | E6056S | 1,920 (20 x 96 samples) | $500.00 | $0.26 |
| Sequencing | R10.4.1 flow cell | ONT | FLO-MIN114 | 96 | $800.00 | $8.33 |
| Sequencing | (Optional) Flow Cell Wash Kit | ONT | EXP-WSH004 |  | $115.00 |  |
|  |  |  |  |  | **Total** | **$25.39** |

**Supplementary Fig. 1. Overview of (A+T)-content and homopolymers (length 3 or greater) in all six amplicons.** Top panels: A+T% in 5bp sliding windows (blue shade) and homopolymer length (purple line); bottom panels: nucleotide composition. A+T% and homopolymers are based on the 3D7 reference strain sequences.


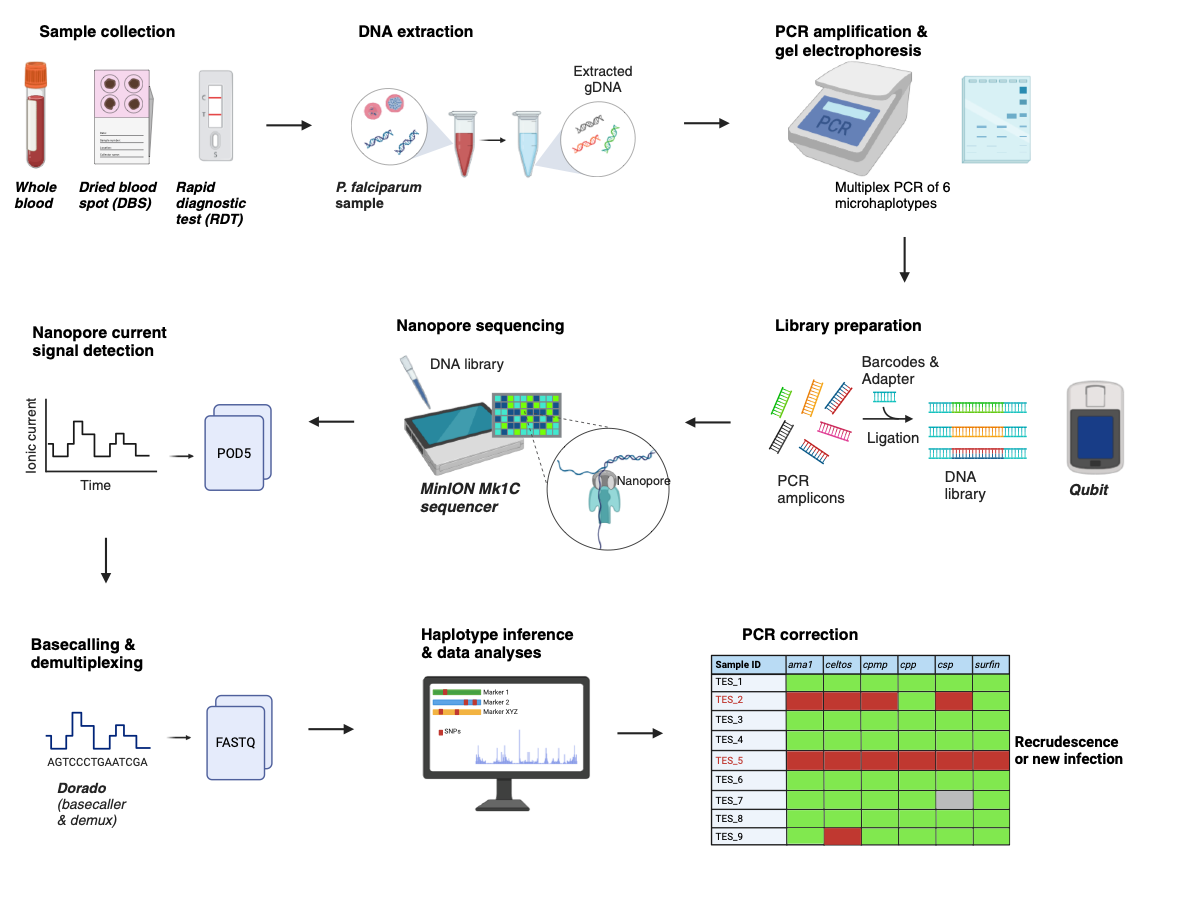


**Supplementary Fig. 2. Overview of the P. falciparum multiplexed nanopore AmpSeq approach using Oxford Nanopore Technologies (ONT).** DBS and RDT samples have been successfully sequenced using the 6-plex microhaplotype panel, however this is dependent on parasite density ^2^. The MinION Mk1B or Mk1D device could also be used instead of the MinION Mk1C. Note that gel electrophoresis is optional, for example if microscopy or qPCR confirmed clinical samples with high parasitemia are used. However, we recommend evaluating the amplification success in parasitemia is unknown or lower parasite density samples are used. Created with BioRender.com.

**Supplementary Fig. 3. Overview of the bioinformatics workflow for haplotype inference of microhaplotypes using ONT sequencing data.** Different software and packages used are shown on the right. Created with BioRender.com.

**Supplementary Fig. 4. Coverage profile of target amplicons using different primer concentrations and two PCR annealing temperatures.** (**a**) First primer-balancing sequencing run using 8 different conditions (4 different primer pool concentrations and 2 different annealing temperatures). (**b**) Second primer-balancing sequencing run using two additional primer pool concentrations, both at 56 ºC annealing temperature. Condition 09 was selected (primer pool new4), highlighted in red, showing most even coverage across all 6 amplicons. All conditions were tested in duplicate using P. falciparum strain 3D7 at 1,000p/μL. Primer concentrations of different pools are shown in Supplementary Table 6. The box bounds the IQR divided by the median, and Tukey-style whiskers extend to a maximum of 1.5 × IQR beyond the box.

**Supplementary Fig. 5. Number of reads and WSAF for true-positive and false-positive haplotypes.** (**a**) Number of reads and (**b**) proportion of reads of the control mixtures across two independent sequencing runs. Only 1 incorrect haplotype was called above the cut-off of ≥20 reads per haplotype (dotted line) in Run 1. Most incorrect reads were contaminating haplotypes (i.e., true-positive haplotypes originating from any other P. falciparum strain present in another sample) and not false-positive haplotypes (i.e., amplification or sequencing errors). Importantly, all but one false-positive haplotypes were below a WSAF 0.1%. 1/1392 (0.07%) in Run 1 and 7/1392 (0.5%) in Run 2 of TP haplotypes fall below the ≥20 reads per haplotype cut-off. The dotted line indicates the minimum coverage of ≥20 reads per haplotype (**a**) and the WSAF threshold of 0.1% (**b**). Note: no cut-off was applied to the data for haplotype inference, meaning all haplotypes (i.e., all reads) identified are shown. The box bounds the IQR divided by the median, and Tukey-style whiskers extend to a maximum of 1.5 × IQR beyond the box.

**Supplementary Fig. 6. Reproducibility in control mixtures. (a)** Inter-assay and intra-assay reproducibility. Inter-assay reproducibility is the percentage of pairs of technical replicates in agreement at the LOD. Intra-assay reproducibility is the percentage of samples for which the three technical replicates agree at the LOD. **(b)** Observed WSAF in laboratory mixed controls of known expected WSAF in two sequencing runs. A postprocessing filtering threshold for minor alleles of ≥ 0.1% and a minimum of ≥20 reads per haplotype was applied.

**Supplementary Fig. 7. Frequency of individual SNPs of the six microhaplotypes in the 20 pre-treatment samples.** Non-reference allelic frequencies (with regard to the 3D7 reference) of each SNP are shown.

**Supplementary Fig. 8. Multiplexity of infection (MOI) of the 20 pre-treatment samples.** MOI was estimated naively (i.e., highest number of alleles in any of the six microhaplotypes) and with THE REAL McCOIL categorical method. Each dot represents one of the 20 samples. Mean MOI was 1.9 for naïve MOI and 1.95 for MOI by THE REAL McCOIL. The box bounds the IQR divided by the median, and Tukey-style whiskers extend to a maximum of 1.5 × IQR beyond the box. Difference in MOI between the two methods was examined using Wilcoxon signed rank test. P value is indicated.

**Supplementary Fig. 9. Number of concordant haplotypes of three markers between nanopore and Illumina sequencing.** UpSetR plots showing the numbers of haplotypes shared between the two different sequencing technologies and different amplicons for the genes (**a**) cpmp (PF3D7_0104100), (**b**) cpp (PF3D7_1475800), and (**c**) csp (PF3D7_0304600). Haplotypes that were found with both methods are highlighted in green. Sequences obtained by Illumina sequencing were trimmed to the same length of markers used for nanopore sequencing.

**Supplementary Text**

*Library preparation and sequencing*

Library preparation was performed using ONT kit SQK-NBD114.96 following the manufacturer’s protocol (version NBA_9170_v114_revL_15Sep2022) with some modifications. Briefly, in the “end-prep” step, the incubation time was increased from 20ºC for 5 min and 65ºC for 5 min to 15 min for each step. End-prepped DNA was bead-purified using 1.6X ratio AMPure XP beads (Beckman Coulter), resuspended in 15 μL nuclease-free water, and quantified on a Qubit 4 fluorometer (ThermoFisher). An equimolar mass of each sample to be barcoded was taken forward into the “native barcoding ligation”. For the “native barcoding ligation”, 3.75 μL normalized (i.e., equimolar) end-prepped DNA was used instead of 0.75 μL, the incubation time was increased from 20 min to 30 min, and barcoded samples were bead-purified using 1X ratio AMPure XP beads instead of 0.4X. For the “adapter ligation and clean-up” step, the incubation time was increased from 20 min to 30 min and the pooled barcoded samples were bead-purified using 50 μL AMPure XP beads instead of 20 μL. Short fragment buffer was used for the wash steps. The final pooled library was quantified on a Qubit fluorometer (ThermoFisher), diluted in elution buffer (ONT) to a total of ~250 fmol, and loaded onto R10.4.1 flow cells. Sequencing was performed on a MinION Mk1C instrument (ONT) with MinKNOW software (version 24.06.15). Three negative controls, consisting of nuclease-free water, were included in each run and underwent the entire workflow, including PCR and nanopore library preparation. A positive control (FCB1) was included in the paired patient samples. An overview of the entire workflow is shown in Supplementary Fig. 1.

*Bioinformatics*

Raw nanopore data (*.pod5 files) were simplex basecalled with *dorado* (v0.8.2; https://github.com/nanoporetech/dorado) using the super-accurate (sup) model (dna_r10.4.1_e8.2_400bps_sup@v5.0.0). To ensure high-quality data for haplotype inference, the minimum q-score for passing reads was set to a stringent value of 20 to minimize erroneous reads (--min-qscore 20 flag; accuracy of ≥99%), as previously described ^2^. Raw reads passing quality filtering criteria were then demultiplexed by barcode using *dorado* (v0.8.2) for native barcodes with the --barcode-both-ends flag set, i.e., double-ended demultiplexing to reduce false positives. A read summary was created using *dorado* (v0.8.2) with the summary command from all basecalled data, i.e., without setting the --min-qscore 20 flag, and quality metrics were evaluated with NanoStat using the --summary option.

From the resulting fastq files, haplotypes were inferred using R packages HaplotypR (v0.5; https://github.com/lerch-a/HaplotypR/releases/tag/v0.5) ^5^ and DADA2 (v1.26.0) ^6^ as previously described with minor modifications ^2^. Briefly, each sample was demultiplexed by marker using demultiplexByMarkerMinION() from HaplotypR. Reads were then filtered for ambiguous base calls (e.g., N) and incorrect sequence length (e.g., to remove amplification and sequencing errors caused by homopolymer-rich regions). Haplotypes were inferred using the function createFinalHaplotypTableDADA2(), with custom ‘OMEGA_A=1e-120’ and ‘pool=pseudo’ settings for highest precision and sensitivity. To further remove false-positive haplotypes the following cut-off criteria were implemented: (1) each marker required a minimum of 1000 total reads per sample, (2) haplotype calls required a minimum coverage of ≥20 reads per haplotype, and (3) a within-sample allele frequency (WSAF) of ≥0.1%. An overview of the bioinformatics workflow is shown in Supplementary Fig. 2. Bioinformatics pipeline and code used for haplotype inference can be found at https://zenodo.org/doi/10.5281/zenodo.14176815. The source code for software HaplotypR, including a tutorial with example datasets, is available at https://github.com/lerch-a/HaplotypR/releases/tag/v0.5.

**References**

1. Schnoz, A., Beuret, C., Concu, M., Hosch, S., Rutaihwa, L. K., Golumbeanu, M., & Nsanzabana, C. (2024). Genotyping methods to distinguish Plasmodium falciparum recrudescence from new infection for the assessment of antimalarial drug efficacy: an observational, single-centre, comparison study. *The Lancet. Microbe*, *5*(11), 100914.
2. Holzschuh, A., Lerch, A., Fakih, B.S., Aliy, S.M., Ali, M.H., Ali, M.A., Bruzzese, D.J., Yukich, J., Hetzel, M.W. and Koepfli, C., 2024. Using a mobile nanopore sequencing lab for end-to-end genomic surveillance of Plasmodium falciparum: A feasibility study. *PLOS Global Public Health*, *4*(2), p.e0002743.
3. Tessema, S.K., Hathaway, N.J., Teyssier, N.B., Murphy, M., Chen, A., Aydemir, O., Duarte, E.M., Simone, W., Colborn, J., Saute, F. and Crawford, E., 2022. Sensitive, highly multiplexed sequencing of microhaplotypes from the Plasmodium falciparum heterozygome. *The Journal of infectious diseases*, *225*(7), pp.1227-1237.
4. Gerlovina, I., Gerlovin, B., Rodríguez-Barraquer, I. and Greenhouse, B., 2022. Dcifer: an IBD-based method to calculate genetic distance between polyclonal infections. *Genetics*, *222*(2), p.iyac126.
5. Lerch, A., Koepfli, C., Hofmann, N. E., Messerli, C., Wilcox, S., Kattenberg, J. H., Betuela, I., O'Connor, L., Mueller, I., & Felger, I. (2017). Development of amplicon deep sequencing markers and data analysis pipeline for genotyping multi-clonal malaria infections. *BMC genomics*, *18*(1), 864.
6. Callahan, B. J., McMurdie, P. J., Rosen, M. J., Han, A. W., Johnson, A. J., & Holmes, S. P. (2016). DADA2: High-resolution sample inference from Illumina amplicon data. *Nature methods*, *13*(7), 581–583.
